# Cook County Hospital

**Published:** 1871-02

**Authors:** Curtis T. Fenn

**Affiliations:** Chicago


ARTICLE VII.—*Cook County Hospital.* Reported by CURTIS T. FENN, M.D., Chicago.

Oct. 21. Pathological. Johnson. Thrombosis, terminating in death. (Case 11, Nov. report.) Inflammation of the wrists, shoulders, knees, elbows and ankles had resulted in terrible abscess of each of these joints. The destruction of tissue had been enormous. A slight murmur over the cardiac orifices had been detected soon after his admission. The inference had been that there existed a thickening of the valves, it might be from fibrinous deposit. On *post mortem* examination, it had appeared that the right cavity of the heart was filled with a colorless clot, extending into the branches of the pulmonary artery, showing that fibrine had coagulated without entangling blood corpuscles. The question had been, what produced the clot? Such clots were one of the acts of death; but they might be found during the last few days of life. A true mechanical coagulum might occur by the roughening of an orifice. Endocarditis might give rise to such effusion as to entangle fibrine. Evidence of acute inflammation of the lining membrane of both sides had been found. There was a morbid point on one of the mitral valves where adhesion had taken place. Probably this had been the point of departure for little clots, which had been swept off by the current of the blood and had been carried to the periphery of the circulatory system where they had become arrested. They had plugged the small arteries, and mortification had ensued.

Specimens. Post mortem pieces of an old man admitted for chronic ulcers of the leg, and partial dementiæ. The urine had not been albuminous. There had been enlargement of the superficial vessels of the abdomen. The ulcers had been cured up, but he had declined in strength, and died. The *liver* was diminished in size, but not in weight. The left lobe was atrophied as it often is in scirrhus. The right lobe cut like a turnip, the resistance being unequal. There were fibrous bands extending into it, so altering its appearance that the specimen might have been taken for anything else as readily as for a liver. There was diminution of the hepatic vessels. A little, pinched, narrowed orifice showed where the vessels passed into the fissure. Cirrhosis of the liver gave rise to abdominal dropsy, but in this case the enlargement of the external abdominal veins had prevented such an arrest of the circulation. This condition was variously described, passing as nutmeg liver or hobnail liver. This man probably had drunk distilled liquor. Germans living to be fifty years of age were apt to have fatty liver. Irishmen had cirrhosis, for they took whisky "*straight*." How did dram drinking produce this liver? If a person took alcohol on an empty stomach, it was speedily absorbed and went to the small vessels as a direct local stimulant; and this, kept up for days, months and years, produced the inflammation. The *kidneys* were somewhat enlarged; there was increase of specific gravity and quite deep congestion. They had become the seat of inflammation from precisely the same cause. The *spleen* weighed 2 lbs. 3 ozs. and 5 drs. The liver being non-distensible, the spleen had received the excess of blood. The *heart* was normal, shown for comparison. No clot. Bands of pleuritic origin formed pockets of membrane between the pleural surfaces. These were the results of a somewhat hasty study of the specimens before us. They would have repaid a further examination.

Surgical. Powell. 1st Case. Stump after reamputation of the thigh. Following the operation, there had been a bulging of the flaps, which increased till the third day, when they were laid open, and a large clot of blood was turned out. On washing the exposed surface, a little bleeding artery had been found and tied. A strong watery solution of carbolic acid had been used. It had whitened the tissues. The flaps then had been drawn together, but not closed. The patient knew too much about the accidents that might come to stumps for his own good. Treatment: beef-tea and opium, with quinine. Opium had a better effect thus by acting on the skin. Lessons to be learned: 1. Never to close till the bleeding has ceased. 2. If hemorrhage has occurred, never to hesitate to open, and turn out and wash. 3. Carbolic acid had been extremely useful, lessening the discharges and promoting their healthy character. The stump had rested on oakum, under carbolized water. After the first two or three days, carbolized water had been omitted on account of the pallor of the edges of the wound, and lessened circulation. No perturbating treatment whatever. The wound was to be dressed simply as possible. Flaps which were left open healed better; they closed up from the bottom. The most successful surgeons did no more than to draw the flaps gently together. If the clothes should stick, we might use a little bit of simple cerate. The bandage would prevent spasm.

Case 2. Another stump. As nearly as could be learned, this man had had a sore of some kind on his thigh, which proceeded from the bone. An attempt to remove it had resulted in a fracture of the femur. The bone had failed to unite. Two months afterwards it had been amputated. The stump had healed; but, six or eight months later, an ulcer had broken out on the end, having the characteristic odor of epithelioma. The ulcer at present was deep, and spreading over the end of the stump; the surface was exceedingly irregular and ragged; the discharge was thin and sanious; the odor was characteristic. The microscope had not detected what, at the present time, was considered a cancer cell—namely, a large cell, with a nucleus corresponding in size and shape, and a nucleolus. But the microscope had lost caste of late in cases like this. If all the microscopes in the world should fail to detect the cancer cell here, he should say it was cancer, from the odor. The removal of cancer had the effect to delay the case from eighteen months to three years, and occasionally it did not return. In thinking of what was to be done, it was best to tell the patient candidly what there would be, namely, a delay of his fate for a few months only. In the meantime, we should use carbolized water as a disinfectant. The hospital had afforded one case of hip joint amputation last year. The subject had died twelve months after the operation, of disease of the lungs. There could not well have been a more unpromising case for the operation than that was. This man would have a good chance if he submitted to a re-amputation at the hip joint.

Case 3. Street car injury. An elderly man, not a laborer. A pretty deep lacerated wound on the inner aspect of the thigh, near the knee, an apparently trivial flesh wound, but really a serious thing. The case presented an angry, bad look. The skin was dead, and gangrenous matter was coming out of the wound. The face was flushed: pulse, 108. Injuries occurring in persons a little irregular in their habits were by far the worst, on account of the danger that gangrene and pyæmia might occur. If this wound were laid open, dead muscle would be found extending far up the thigh. This patient ran two risks; the first from pyæmia, the second from hemorrhage, as the slough would be near the femoral artery. Such a wound should not be stitched. The freer its discharges the better. There was little feeling in the leg, and a decided lessening of the temperature. Treatment: Beef tea, milk, and the stimulant he had been accustomed to. If mortification should occur, free incisions and carbolic acid. Carbolic acid might retard a separation of the slough, but it certainly prevented extensive discharges, from which exhaustion occurred.

This patient presented also a *tumor of the scrotum.* It was not a cancer, nor a sarcocele, nor a hernia, nor a varicocele. It was, evidently a hydrocele. This was a pear-shaped tumor, giving fluctuation and transmitting light. The scrotum should not be laid open in hydrocele; for thereby the cavity of the tunica vaginalis would be destroyed. A large, irregular corrugated cicatrix would remain, and, lastly, cases were cured by injection. If this man survived the leg, the hydrocele might be dealt with.

Oct. 25. Medical. Ross. 1. Case, just admitted, a woman with dropsy of the feet, existing fourteen days. The lecturer said that it must have had its origin in the heart or kidneys. The pulsation of the cervical-veins was noticed. By percussion it was found that the heart extended one and a half inches to the left of the nipple. The impulse was very feeble indeed. A loud, blowing murmur was heard all over the precordia; loud at apex and base, and propagated along the sternum. The murmur was heard over the carotids. The history elicited showed that she had been troubled in her breathing for eight years. She had complained of pain in her shoulder, and of numbness in her left arm. The heart was greatly enlarged and the pulse feeble. It was not hypertrophy, but dilatation. The only medicine indicated was to be directed to the anæmia. No active diuretics or cathartics could be of any avail, for the dropsy had come on in the last stage of her disease, and would be fatal in a little time.

2. Case, of interest in diagnosis. The patient had been admitted one year ago, and was now employed as a nurse. There was great disparity between the two sides of his chest; the left was fully dilated on forced inspiration, while the right was not. There was no effusion of pleurisy, for that enlarges. The respiratory murmur, although weak, was heard all over, and was soft, and of the same quality as that on the other side. There was no cough. He had some difficulty in breathing. On examination of the heart there was found a distinct murmur at the base. Could an aneurism have been the cause? A swelling in the ascending aorta might encroach on the right bronchus. There was a whizzing sound detected over the region of the right bronchus, quite different from that heard at the base of the heart. There was no disturbance of the circulation. It could not, therefore, have been of the transverse or of the descending portions. The patient had been improving by time.

Surgical. Powell. During the last two weeks two cases of hydrocele had been lost to the clinic. They were young men from the country coming to enlist. They had been sent here to be cured, but being told soon after admission that it involved a serious operation they left.

Alluding to the case of a child run over by the street cars. Opium to relieve pain, dry heat, and any means to stop hemorrhage, were the best means to allay shock. He would not give alcoholic stimulants.

1st Case. Compound fracture of the leg. Sailor. A heavy spile struck him from behind. It was observed how all fractures of the tibia are exceedingly oblique, the lower end of the upper fragment always coming forward. Malgaigne had a machine for screwing it down. If prominent in a compound fracture, it would be better to saw it off. A suit for malpractice had been on the docket for the same thing; but it would be good practice. The opening might be enlarged, if necessary, to get the bone back; then we should close the wound carefully, and, if possible, procure a simple from a compound fracture. We should not be afraid of any redness or a little inflammation. It is nature's way of healing. We should not put cold water on, as a rule, for by it we may chill the part and prevent the healing; but a rag may be wrung dry and placed on the wound if the patient desires, for that soon gets warm. The limb should be placed in a fracture box, with sides to be let down for cleaning. No perturbating treatment whatever. Diet of anything he chooses, and opium to relieve pain.

2d Case. Street car injury, seen four days ago. Moribund. The thigh and leg gangrenous and pulseless. Not shown, but cited to illustrate the serious consequences of such injuries.

3. Case of a woman whose toe was amputated some days ago, with a good result, compared with the case of a man who had erysipelas and palmar abscess following amputation of a finger. These results depended on the condition of the patient rather than on the skill of the surgeon.

4. Case of loss of tissue of the neck, resulting from erysipelas. On admission there had been a considerable cavity dissected between the sterno mastoid muscles near their origin. The posterior surface of the base of the sternum had been uncovered. A bad cicatrix remained.

5. Case of a woman with necrosis of the extensor tendons of the hand, from erysipelas after a bite. The result would be a stiff hand, better off than on.

6. Case of acute orchitis, shown one week before; treated by a forty-grain solution of nitrate of silver, which had been applied with a camel's hair pencil, and covered with cotton. The result had been very satisfactory—immediate relief of pain and swelling. These cases, treated heretofore by strapping, would be made to test the merits of nitrate of silver, so much vaunted of late.

7. Case of abscess in the groin, ready to open; presenting all the appearance of a bubo. It is a bubo, but without a specific origin. This man was a coachman; he received a stroke from the shaft of a carriage.

Oct. 28. Pathological. Johnson. Case. Locomotor ataxia, from the outside dispensary. His gait and manner of sitting were observed. He had been taken for a drunkard a hundred times, though he never drank. He was worse at night. There was an unbinding of the organs used in locomotion. The statements of pathologists were widely different as to the changes of structure in this disease. A careful examination as to the consistence and quantity of the blood distributed, the character of the nerve cells, the conditions and relations of these, always showed some disintegration, softening, or degeneration. There was, finally, a molecular change in the muscular fibres themselves. They became wasted in volume. The articulations became loosened, and spontaneous luxation sometimes occurred. A case was cited in which both hips were thus dislocated in one night. Nutrition failed. Atrophy begun, not only for want of use, but for want of that, a supply of which, in a measure, affects nutrition. Some writers attempted to confound progressive muscular atrophy with this disease; but progressive muscular atrophy commenced in the periphery—this in the centre. Progressive muscular atrophy sometimes affected one muscle; this affected the whole system of muscles. This and that were quite different. This was *ab initio* a central disease.

Post mortem specimens. Lungs. Emphysema. Pieces passed. The first thing to be received in pathology was, that every morbid specimen was clean. The air vesicles were large. The lungs did not collapse when an opening was made in the chest. Vesicular emphysema consisted in vesicular dilatation; it might be by general dilatation, by stretching of septæ, or by dissolution of the walls and coalescence of the vesicles. The fact was, that the vesicles coalesced in multitudes, for we saw large bladders of air. It was more apt to begin in the upper and anterior portions of the lung, giving prominence to the inter-clavicular spaces, and producing the barrel-shaped chest. Sometimes there was a dilatation of the bronchial tubes, but these cases were not necessarily connected. The apices were studded with tubercle and hardened masses of cicatrices, also. How was such a case produced? The case was perfectly normal in the conjunction of these two affections. Valieux says that we almost always have some emphysema in tuberculosis. Many authorities denied it, but it was true. The theory of Valieux was, that a tube becoming plugged, the air which is intercepted passes into a neighboring tube, resulting in too great pressure, and the breaking of walls. (Discussion of other theories. Weight of authority said to be balanced.)

Surgical. Powell. Case of a Dutchman, who, with his wife, had been run over by the cars at a railroad crossing. Admitted in bad condition, with a compound fracture of the leg. He had been a long time poised between life and death. While waiting for a better condition to arrive before amputation, Dr. Powell had performed a little operation of conservative surgery. The ends of the bones, badly necrosed and overriding, giving rise to a large amount of suppuration, had been cut off, and an attempt had been made to press the fragments down more nearly in apposition; but they could not be kept in place, so the bones were left to themselves. Finally, the leg had been put in a starch bandage, and the man sent home. To-day he came back well as ever. Shown to illustrate what can be done by a little perseverance and conservative treatment.

Nov. 1. Medical. Ross. Case 1. Cancer of the liver. The patient having been sick almost a year came into the Hospital with a large tumor in the abdomen, and a cough of a peculiar character. It had been concluded, from the cachexia, nodular swelling, and great pain in the liver, that it was cancerous. Pain was a symptom in cancer of the liver which distinguished it from waxy liver. A few days ago he had been taken with diarrhœa, and bloody stools, followed by great diminution of the tumor. The case would prove rapidly fatal.

Case 2. Typhoid fever, taken three weeks ago, shown on account of great tympanitis. The pulse had been as high as 140, and there had been great prostration and accompanying delirium. The patient had improved under alcoholic stimulants, beef tea, and milk. There was tympanitic resonance all over the abdomen, and a good deal of tenderness in the right iliac fossa. The tympanites in this case produced pressure on the diaphragm. It sometimes oppressed the circulation. It was important to look out for this. He had taken turpentine emulsion to control diarrhœa. Cloths sprinkled with turpentine had been applied over the abdomen. Sometimes, enemas, containing a little of the oil, had been administered. He had likewise taken 1-32 of a grain of strychnia, 2 grains of quinine, and 3 drops of nitric acid, three times daily. The tongue was still dry and fissured.

Case 3. Remittent fever. Without entire intermission, the fever was generally better at night and early morning. The treatment was very simple, the great matter being diagnosis. Sometimes great irritability of the stomach was allayed by calomel, but this symptom was caused by the fever. Give quinine in large doses, and frequently. We should not wait, but give quinine during the fever, in order to lessen, and finally to break it. If the symptoms should be modified while the fever continued, then we might treat as we would continued fever. There were a few cases that could not be cured at once with quinine; but, notwithstanding the blood was so changed, if these cases were taken in time, quinine would cure them. Palliatives, cold to the head, neutral mixture to allay irritability, and opium at night. Sometimes the stomach would not bear acid. Then we should give quinine without acid. In this case the fever was broken. Quinine needs to be continued in small quantities three times daily.

Case 4. Typho-malarial fever. Convalescent. When admitted he had presented the appearance in every particular of remittent fever. In the course of a little time there had come up every symptom of typhoid. It had commenced as malarial, and seems to have had grafted upon it typhoid. After the treatment of remittent fever, we should give the acid treatment of typhoid, in such cases.

Case 5. Acute Bright's disease. Negro. When last shown he had been convalescent, while taking a little jalap and cream of tartar and a hot air bath daily, but from some cause he had got worse. The fever became high, the skin hot and dry, urine suppressed; terminating in uremic convulsions, and coma. The drawing of thirty ounces of blood had given him complete relief. This was an instructive case. It had been impossible to administer medicine. He had been saved by bleeding. Blood-letting was the great remedy in uremic convulsions. This patient had had an active cathartic as soon as it became possible. He immediately had seemed about as usual before the relapse. Since then, hot air baths and mild laxatives had been resumed. That linen shirt which he was wearing might have been the cause of his taking cold, though every precaution had been taken to protect him after his baths. He ought to have been dressed in flannel. He was now almost well again.

Case 6. Opium eating. A slender youth. He used to take two grains of morphia at a time four years ago, and finally twenty-five grains of opium had been required to meet his longing. At last he had tried to break off. He had been sent here from the Bridewell for malingering. He had not had any opium for four or five days. Shortly before his admission he had been taking ten grains of opium three times a day. He now took thirty grains of bromide of potassium, four times a day. He said he felt a sinking and gone sensation in his stomach when deprived of his accustomed allowance. We should be on our guard against opium or morphia in chronic disease. The use of the bromide of potassium would be continued, for that was a remedy which could be broken off with greater ease. It relieves him of his nervousness.

Case 7. Typhoid fever, complicated with broncho-pneumonia. The injected condition of the cheeks, generally present in pneumonia, observed. There was dullness on percussion over the lower portion of the right lung. No very active treatment could be had for such complications. We might, besides the treatment for typhoid, use a poultice or else oil silk and flannel. Afterward if anything is done, we might give a stimulant expectorant, as the carbonate or the muriate of ammonia. Sometimes quinine, sanguinaria, and Dover's powder—nothing depressing.

Surgical. Powell. Case 1. Re-amputation of the thigh at the hip joint. This subject was introduced to the clinic one week ago, as a case of cancerous ulceration of a stump of the thigh, about a year after amputation, for supposed necrosis of the femur, (Case 2, Oct. 2.) He had borne the former operation exceedingly well. A good result again was expected, and through it a prolongation of his life. The abdominal tourniquet was not considered at all necessary, though it had served a useful purpose. The artery might be compressed with the thumb at either the umbilical or iliac region. A great variety of operations were mentioned. The anterior and posterior flap method was recommended as suiting best. There were objections to it, however. Celerity was no object since the introduction of anæsthetics. Last winter the operation had been performed in forty-five seconds. The subject, a small man, cachectic, aged about forty, was given a few swallows of whisky and the operation was commenced. Chloroform was administered by the house physician and his assistant. Dr. C. T. Parkes controlled the circulation by digital compression of the aorta. Dr. Ben. C. Miller took the stump. There was no trepidation or haste; deliberately but surely the different steps of this trying operation were taken. The shock and exsanguination were profound. Reaction took place, and recovery began.

Nov. 4. Pathological. Johnson. Specimen of malignant disease of the liver. Weight of the liver, 12 lbs., 10 oz. Sometimes nodules were felt beneath the ribs; not always. Here they were perceptible by taking hold of the border of the liver and pinching it. There had been œdema of the lower extremities, but no ascites. The hepatic vessels had been probably unimpeded. When cut into, the liver had a yellowish appearance. It required the microscope to make out the nature of these changes. It looked like a fatty liver. Some spots had decidedly the appearance of cirrhosis. Microscopic examination of the juices, scraped from a cut surface, discovered an abundance of those forms peculiar to cancerous growth. What constituted a cancer cell? 1. A cancer cell was a large cell, relatively, 1-2000 to 1-500 of an inch in diameter. It had no peculiar form, it was spheroid, ovoid, caudate, stellate, fusiform. 2. It has one or more nuclei, relatively large also. Epithelial cells had small nuclei. A perfectly formed cell was rarely to be met with. 3. A nucleolus. All highly refractive. Granular matter was always formed in the cell, in the nucleus and around the nucleolus. The microscope was not always available. In certain forms of cancerous tumors a large cell could not be found. In such cases the clinical history of cancer must be obtained. In the specimen considered there were free nuclei in excess. Nuclei and nucleoli were subject to great diversity in size. The presence of these free nuclei in excess, with the clinical history, furnished infallible evidence of cancer. Clinical history had to be taken into consideration in other cases, studied very satisfactorily by means of physical exploration. The microscope furnished just the kind of evidence that the stethoscope and pleximeter did. It might decide operations. If the physical signs in diagnosis are not neglected, neither should the revelations of this invaluable instrument be ignored.

Specimens. 1. Aneurism of the ascending arch of the aorta. Death, one year ago, caused by rupture into the œsophagus. The stomach was full of blood at the autopsy, and the patient had vomited three pints just before expiring. This tumor had compressed the left bronchus, so that the respiratory murmur was absent except in forced respiration. Pressure on the œsophagus made deglutition difficult, but he could swallow solids better than liquids,—a somewhat unusual fact. Rupture into the œsophagus occurred in seven per cent. of such cases of aneurism. The well preserved wall showed the characteristic laminæ of fibrine. The true cardiac sounds had been transmitted through the sack as through a stethoscope.

2. Dilatation of the arch of the aorta, a great constriction existing further on at a point where there had been atheromatous degeneration. The constriction was now the seat of calcification. This was a dilatation and constriction, not an aneurism. The semilunar valves of the left side were roughened and thickened by the same degeneration. As the blood passed, there had been aortic regurgitation and a direct or obstructed aortic murmur, heard down to the fourth lumbar vertebra. These murmurs were sometimes heard at a very great distance.

Surgical. Powell. The man on whom the class saw him perform the operation of amputation at the hip joint four days ago, was in a favorable condition to-day. Pulse about 80; discharge favorable. The surfaces exposed by the operation had been touched with carbolic acid. Two things had followed: little discharge and little pain, for carbolic acid was a local anesthetic, as all who had spilled it on their fingers knew. The patient was kept quiet; light diet, as much beef tea as he could take, and a bottle of wine at his side from which he drank as he was inclined. The man was exceedingly cheerful and happy.

Case 1. From the Dispensary. A young man who began, four years ago to notice pain in his right side at night, a burning pain across the lower part of the bowels, and frequent micturition at night, "six or seven times inside of a minute." Since then he had had gonorrhœa. He said he had been sun-struck two years ago. He had lost flesh, had had pain in his head, back, and limbs. The symptoms were chiefly referable to the genito-urinary organs. Such cases must be examined very carefully. We should run over in our mind what it might be, cystitis, calculus, prostatitis. Prostatitis hardly ever occurred in a young person. Examine the urine; sound and discover the seat of pain. Speaking of the difficulty of detecting stone at all times, he cited the case of a gentleman from Minnesota; his brother, who was a physician, had accompanied him to Philadelphia, consulting the most eminent surgeons there. Thorough search for supposed calculus had been made by them all. No satisfaction had been obtained. He had received their written opinion to the effect that no calculus existed. He had been examined here likewise, afterward. It was an extremely aggravated case. Dr. Powell had been convinced that he detected a stone enfolded in mucous membrane upon the anterior surface of the bladder. An operation had proved it. He had removed a very large calculus from that situation. The man had been cured and sent on his way rejoicing. The case was cited here, to illustrate one of the difficulties of diagnosis. As to the case before us, if it were cystitis there would be great pain. We should be careful in the use of the sound, using no force. It had been thrust through the walls of the bladder. A little finesse and an acquaintance with the anatomy of the part were required. There might be a stricture and cystitis in consequence—here the sound was introduced—once in the bladder it produced no pain, but the patient complained bitterly when the point of the instrument arrived at the neck. No stone was found. Soreness at the neck of the bladder the only thing of which information was gained. The patient would be admitted to the Hospital, and the case would be deferred for further examination. Treatment: warm hip baths, and a sound passed and allowed to remain fifteen minutes each day.

Case 2. Ingrowing nail. A lad who had been wearing a tight boot. These cases were troublesome and painful. If the nail should be cut, the deformity would be made worse by it. A large number got well by letting alone, the nail finally riding up out of its place in the flesh. Operation: Chloroform; a portion of the nail extirpated with knife and scissors.

Case 3. Result after amputation of the leg. Would some one of the class tell whether this was a circular or a flap amputation? (Answers did not agree.) It was a circular. Flap amputations were longer in getting well; suppuration was greater, and there was more danger of septæmia. The fault with this had been, a little too short a flap of integument and obliquity of the cut surface of the bone. Still it only needed a little time to result in a fine stump.

Case 4. Swelling in the groin, large, almost diffuse, some little evidence of suppuration. The patient had not had syphilis. As a rule, the statements of persons of this kind may be taken, for, if they have had syphilis, they do not hesitate to acknowledge it.

Case 5. Brought in, both walking and looking like a drunken man, but he was not intoxicated. His horse had run away with him that day. When picked up he had vomited quite a quantity of blood; he had vomited, too, as he came in. He was laboring under some serious injury. Any lesion of the head, a slight scratch, might be followed by grave symptoms. A case had lately occurred at the Rolling Mills, in which paralysis, coma, and death supervened ten days after a slight injury to the head. But there had been cerebritis all that time. This man would be allowed time for reaction, and would be shown hereafter.

Case 6. Of palmar abscess or felon. A young woman. The only safety was in making a free, early incision. If only little punctures were had, there would be burroughing instead of escape of pus.

